# Whole-genome sequencing revealed concurrent outbreaks of shigellosis in the English Orthodox Jewish Community caused by multiple importations of *Shigella sonnei* from Israel

**DOI:** 10.1099/mgen.0.000170

**Published:** 2018-03-27

**Authors:** Vanessa Rew, Piers Mook, Suzan Trienekens, Kate S. Baker, Timothy J. Dallman, Claire Jenkins, Paul D. Crook, Nicholas R. Thomson

**Affiliations:** ^1^​ Public Health England, London, UK; ^2^​ University of Liverpool, Liverpool, UK; ^3^​ Sanger Institute, Hinxton, UK

**Keywords:** *Shigella sonnei*, outbreak, Orthodox Jewish Community

## Abstract

In December 2013, Public Health England (PHE) observed an increase in the number of cases of *Shigella sonnei* linked to the Orthodox Jewish Community (OJC). Ultimately, 52 cases of *S. sonnei* phage type (PT) P and PT7 were notified between November 2013 and July 2014. Whole-genome sequencing (WGS) was performed on a HiSeq 2500 platform (Illumina) on isolates of *S. sonnei* submitted to PHE during the investigation. Quality trimmed sequence reads were mapped to a reference genome using BWA-MEM, and single-nucleotide polymorphisms (SNPs) were identified using GATK2. Analysis of the core genome SNP positions (>90 % consensus, minimum depth 10×, MQ≥30) revealed that isolates linked to the outbreak could be categorized as members of distinct monophyletic clusters (MPCs) representing concurrent regional outbreaks occurring in the OJCs across the United Kingdom. A dated phylogeny predicted the date of the most recent common ancestor of the MPCs to be approximately 3.1 years previously [95 % highest posterior density (HPD), 2.4–3.4]. Isolates of *S. sonnei* from cases from the OJCs in Israel included in the phylogeny, branched from nodes basal to the UK OJC outbreak clusters, indicating they were ancestral to the UK OJC isolates, and that the UK isolates represented multiple importations of *S. sonnei* into the UK population from Israel. The level of discrimination exhibited by WGS facilitated the identification of clusters of isolates within the closely related bacterial populations circulating in the OJC that may be linked to a unique point sources or transmission routes, thus enabling a more appropriate public health response and targeted interventions.

## Data Summary

Short-read FASTQ sequences have been deposited in the NCBI Short Read Archive under the BioProject PRJN A248042 (http://www.ncbi.nlm.nih.gov/bioproject/248042). The accession numbers are listed in Table S1 (available in the online version of this article).

## Outcome

The level of discrimination exhibited by WGS facilitated the identification of clusters of isolates within the closely related bacterial populations circulating in the OJC that may be linked to a unique point source or transmission route, thus enabling a more appropriate public health response and targeted interventions.

## Introduction

Species of the genus *Shigella*, including *Shigella dysenteriae*, *Shigella boydii*, *Shigella flexneri* and *Shigella sonnei*, are the most common cause of bacterial dysentery (bloody diarrhoea) worldwide. Although all species of the genus *Shigella* contribute to the high burden of diarrhoeal disease in low-income regions, *S. sonnei* is the most commonly reported species in middle- and high-income countries [1].

In England an average of approximately 1100 laboratory-confirmed *S. sonnei* cases are reported by all local hospital laboratories to the national Second Generation Surveillance System (SGSS) of Public Health England (PHE) each year. Referral to PHE is recommended but not mandatory. Approximately 800 (73 %) of the 1100 isolates reported by all local hospital laboratories from faecal specimens from hospital or community cases with symptoms of gastrointestinal disease are sent to the PHE for confirmatory bacterial identification and typing.

Until now the detection and investigation of outbreaks of gastrointestinal disease caused by *S. sonnei* has been hampered by the lack of a robust typing method. *S. sonnei* is defined by a single somatic (O) antigen and so cannot be further subtyped based on serotype. Traditionally, phage typing has also been used to differentiate isolates, but this method has low discriminatory power and is a weak predictor of phylogenetic relationships between isolates [2]. Recently, whole-genome sequencing (WGS) has been used to define the population structure of *S. sonnei* at the global level [3]. Furthermore, the analysis of single-nucleotide polymorphism (SNP) typing has been shown to be a robust and highly discriminatory subtyping technique for the detection and investigation of outbreaks of *S. sonnei* at the local and regional level [4, 5, 6].

Since the 1980s, outbreaks of *S. sonnei* have been described in the Orthodox Jewish community (OJC) in England and elsewhere [7–9]. In December 2013, the North East and North Central London Health Protection Team (NENCL HPT), PHE observed an increase above the expected number of cases of *S. sonnei* phage type (PT) P and PT7 among residents of north London records on SGSS for this location, compared with previous weeks and the same time frame in previous years [10]. Epidemiological information linked to these cases indicated that the cluster of cases included members of the OJC (Charedi and other OJCs) living in different parts of north London.

The number of confirmed *S. sonnei* cases in London continued to increase during 2014, with two cases also being identified in the Greater Manchester area. The Outbreak Control Team (OCT) requested that the isolates were sent for molecular typing to facilitate ongoing outbreak investigations. Based on previous *S. sonnei* outbreak work in this community in London [5] multi-locus variable number tandem repeat analysis (MLVA) [11] has been shown to have limited value in discriminating isolates of *S. sonnei* in this context and WGS was adopted as the molecular typing method of choice. The aim of this study was to describe the outbreak investigation and highlight the added insights that the prospective use of WGS provides in this context, including whether these cases occurred as a result of continuous transmission within the UK from previous outbreaks or more recent importations from elsewhere.

## Methods

### Epidemiological investigation

In response to the increase in the number of *S. sonnei* PTP and PT7 in the London area in December 2013, an epidemiological investigation was initiated by NENCL HPT. Having reviewed the geographical distribution of these isolates, a formal definition of a case for the epidemiological investigation was considered to be a resident of the London boroughs of Barnet, Hackney or Haringey with confirmed *S. sonnei* infection, belonging to PTP or PT7, notified to the NENCL HPT between November 2013 and July 2014. Laboratory notifications were monitored and local environmental health departments were requested to send exposure questionnaires (collecting information on recent travel history, school attendance, occupation and food consumption) to all cases and return completed questionnaires to the NENCL HPT. Information on patient’s case history and clinical presentation were collected from clinicians when notifications were made by phone. Data were collated and descriptive analysis was performed.

### Bacterial isolates

WGS from 107 isolates from cases linked to the current Barnet/Hackney/Haringey outbreak, and from historical outbreaks in the OJC in England in previous years (2008=9; 2009=8; 2010=9; 2011=26; 2013=12; 2014=43) [12] were analysed during this study. Of the 26 isolates collected in 2011 24 were from an outbreak previously described by McDonnell *et al*. [5]. Also included were 29 isolates from the Barnet/Hackney/Haringey outbreak, collected in 2013 and 2014 (see above; Summarised in Table S1). All isolates included here were phage typed using the method described by Bentley *et al*. [13] (Table S1).

To add context an additional 238 publicly available whole-genome sequences from patients linked to members of the OJC living in Israel (*n*=216), France (=16), Belgium (*n*=3) or the USA (*n*=3) [12] were also included in this analysis.

### Whole genome sequencing

DNA was extracted using the automated QiaSymphony platform (Qiagen). Tagged multiplexed genomic libraries and DNA sequencing was carried out using a HiSeq 2500 platform (Illumina), at PHE and the Sanger Institute as described previously [14, 15].

Illumina reads were mapped to the reference *S. sonnei* strain Ss046 chromosome (EMBL ID: CP000038) [16] using BWA-MEM [17]. The Sequence Alignment Map output from BWA was sorted and indexed to produce a Binary Alignment Map (BAM) using Samtools [18]. GATK2 [19] was used to create a Variant Call Format (VCF) file from each of the BAMs, which were further parsed to extract only single-nucleotide polymorphism (SNP) positions which were from high quality genomes (MQ>30, DP>10, Variant Ratio>0.9). After recombination detection by Gubbins v1.3.3 [20] and masking of detected recombinant regions a final set of 5418 polymorphic positions were used to infer maximum-likelihood trees using RaxML v8.1.17 [21].

Timed phylogenies were constructed using BEAST-MCMC v1.80 [22]. Alternative clock models and population priors were computed and their suitability assessed based on Bayes Factor (BF) tests. The model with the highest support was a relaxed lognormal clock rate under a constant population size. All models were run with a chain length of 1 billion. A maximum clade credibility tree was constructed using TreeAnnotator v1.75 [22].

## Results

### Epidemiological investigation

**Fig. 1.**
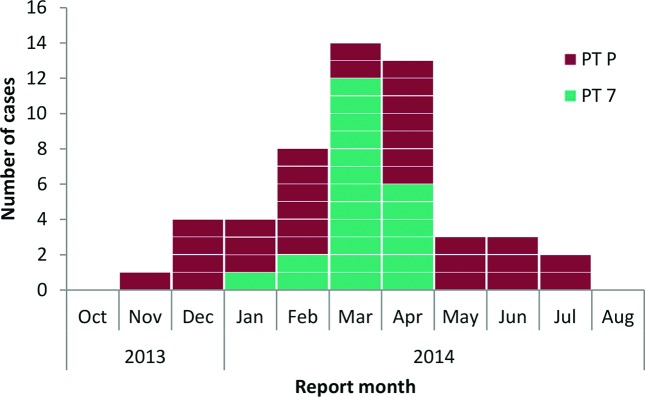
Epidemic curve showing confirmed cases of *S. sonnei* (phage types P and 7) in residents of the London boroughs of Barnet, Hackney and Haringey notified between 12 November 2013 and 10 July 2014 (*n*=52).

**Fig. 2.**
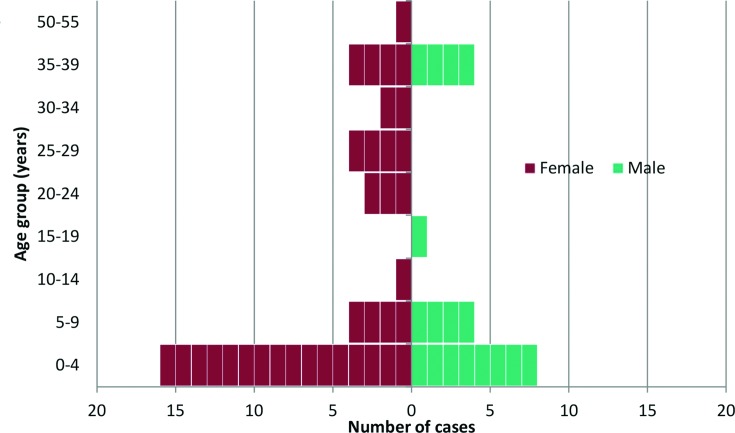
*S. sonnei* phage type P and 7 according to age band (years) and gender notified between 12/11/2013 and 10/07/2014 (*n*=52).

A total of 52 patients presenting with confirmed *S. sonnei* PTP or PT7 derived gastrointestinal disease were notified to PHE between 12 November 2013 and 10 July 2014 (Fig. 1). The peak number of cases was reported in March and April 2014 (*n*=14 and *n*=13, respectively). Patients with a confirmed *S. sonnei* infection belonging to PTP were observed for the duration of the outbreak period, with the peak number of reports in February and April 2014 (*n*=6 and *n*=7, respectively). *S. sonnei* PT7 cases were reported between January and April 2014 only and peaked in March (*n*=12). The outbreak was declared over on 28th July 2014, at which point two incubation periods (two weeks) had passed without further notification of a case matching the case definition. From all cases the median patient age was six years (range 0–55; 46 % were aged less than five years) and 67 % were female (*n*=35) (Fig. 2). Of the total number of cases, 60 % were reported from the borough of Barnet (*n*=31; over the duration of the outbreak period), 25 % from Hackney (*n*=13; between January and April 2014) and 15 % from Haringey (*n*=8; between January and June 2014).

Throughout the outbreak period, eight (15 %) patients completed a questionnaire. Additional epidemiological information was gathered on a further 13 (25 %) cases by the Health Protection Team when contacting patients directly, or from speaking to their family or a notifying clinician. Twenty cases (38 %) either self-identified as Jewish via a questionnaire, or identified by their general practitioner as Jewish (of which two stated they belonged to the orthodox Jewish community, but not Charedi), and 28 (54 %) had a traditional Jewish name. One patient was not Jewish but worked as a child-minder for a large family from a ‘religious community’ of which she declined to name. The remaining three cases did not have traditional Jewish names and were not contactable.

Of the cases linked to additional epidemiological information (*n*=21), 10 (48 %) reported living with a household member who had similar symptoms but did not submit a faecal specimen for microbiological testing. Among all of the cases, six were known to have been admitted to hospital (aged between two and 27 years). Only two cases reported a history of travel less than seven days prior to onset of symptoms, one returning from Australia and the other from Israel. The case travelling from Australia reported they were symptomatic on entry back into England.

Two childcare settings and three common households were associated with cases identified during the outbreak period. One of the childcare settings was a girls’ primary school in North West London attended by four patients, all female and aged between 3 and 4 years.

### Molecular typing by WGS

**Fig. 3.**
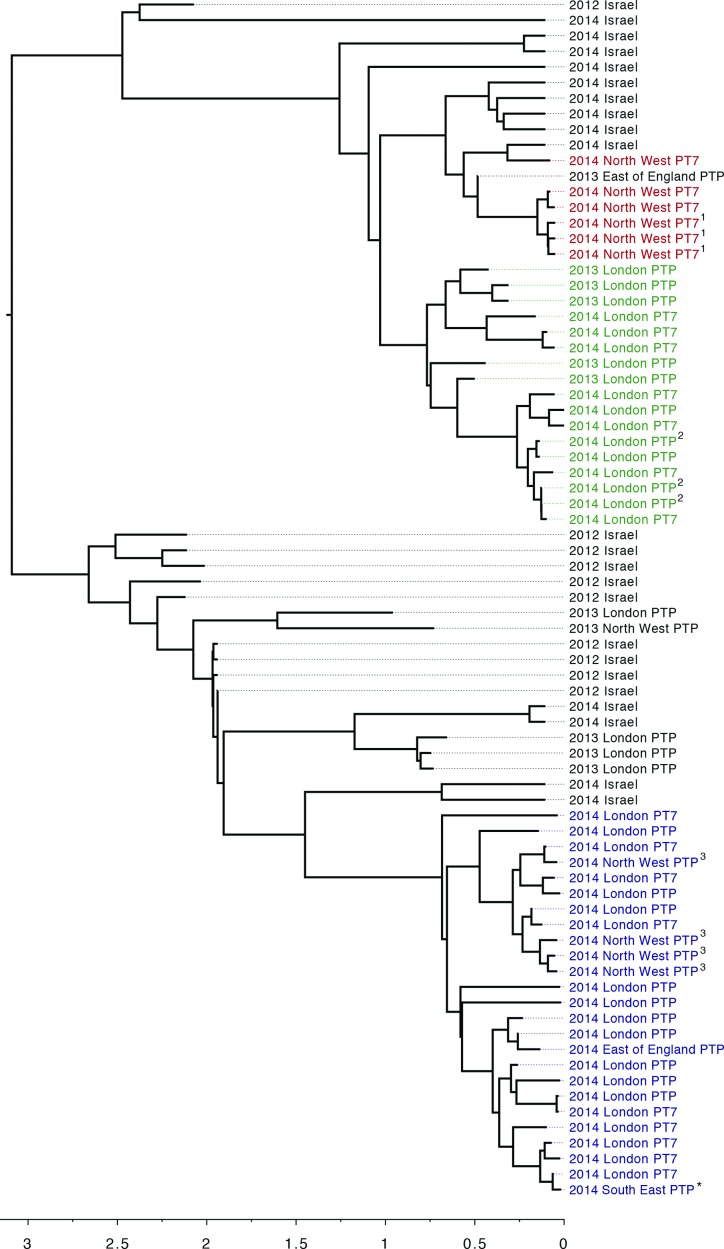
Maximum-likelihood tree of *S. sonnei* strains circulating in OJ communities in England between 2008 and 2014 split into MPCs. MPC1 (red) consisted of six cases from Oldham including three household contacts; MPC2 (green) consisted of 17 cases from the London borough of Barnet, and included four household clusters; and MPC3 (blue) consisted of 25 cases, 19 of whom resided in the London boroughs of Hackney and Harringey. The three household clusters are highlighted by superscript numbers (^1, 2, 3^) The child-minder is highlighted with an asterisk (*).

WGS revealed concurrent regional outbreaks of three monophyletic clusters (MPCs) occurring in the OJCs across the UK (Fig. 3). MPC1 (highlighted red in Fig. 3) comprised six isolates from Oldham (a local authority in Greater Manchester) including three household contacts. Analysis of the WGS data by pairwise comparisons with all the other isolates in this study showed the minimum pairwise difference between isolates in MPC1 was zero SNPs with a medium of one SNP and a maximum of 4 SNPs. A seventh case belonging to MPC1, resident in the East of England, was isolated in 2013. MPC2 (highlighted green in Fig. 3) comprised isolates from 17 cases with minimum, median and maximum pairwise WGS SNP distances of 0, 4 and 11 SNPs, respectively. These cases resided in the London borough of Barnet, 10 of which were cases identified in the epidemiological investigation, and included three household contacts. MPC3 (highlighted blue in Fig. 3) comprised of 25 isolates. Analysis of the WGS data by pairwise comparisons with all the other isolates in this study revealed that the minimum pairwise difference between isolates was zero SNPs with a medium of three SNP and a maximum of six SNPs. Of the 25 cases in MCP3, 19 resided in the London boroughs of Hackney and Harringey and all were identified in the epidemiological investigation, four resided in Oldham, one in the East of England and one in Crawley in the South East of England. There were four household contacts.

By reconstructing a dated phylogeny the date for the most recent common ancestor of the three MPCs was estimated to be approximately 3.1 years previously [95 % highest posterior density (HPD), 2.4–3.4]. The household contacts in MPC1 were located on the same branch but those in MPC2 an MPC3 were not (Fig. 3). In the timed-phylogeny, the posterior probabilities at the terminal nodes within the individual clusters were low, due to insufficient genetic diversity between the isolates. Therefore, with respect to person-to-person spread of infection, the intra-cluster topology may be misleading and not necessarily accurately represent the transmission events.

Placing the OJC outbreak clusters into a broader national and international phylogenetic context indicated that *S. sonnei* circulating in England amongst the OJC between 2008 and 2014 were closely related to each other but distinct from contemporary *S. sonnei* isolates circulating outside of the OJC in England. Instead, they clustered more closely with *S. sonnei* isolated in Israel [2, 5, 12] (Fig. S1). Strains in the PHE archive isolated from OJC cases could be differentiated in time, with isolates from 2008, 2009, 2010–2011 and 2013–14 clustering on different branches within the OJC phylogeny (Figs 3 and S1). These data also indicated that lineages taken from Israel were interspersed with the three English OJC outbreak clusters, with the isolates from each of the three outbreak clusters described in this study being more closely related to concurrent and historical isolates from Israel than to each other and other *S. sonnei* isolates in neighbouring clusters (Fig. S1).

Over the past 20 years, strains of *S. sonnei* associated with the OJC in the UK have been typed as PTP. This outbreak, included strains belonging to PTP and PT7 (Fig. 3 and Table S1). These PTs were not associated with a phylogenetic signal within the OJC cluster; isolates with the same core genome had different PTs and strains on distant branches of the phylogenetic tree had the same PT among national isolates and those in identified in the epidemiological investigation with WGS results (Table 1).

**Table 1.** Distribution of phage type P and 7 among isolates of *S. sonnei* within MPCs

	Total in MPC (*n*=48)		Outbreak-related isolates in MPCs (*n*=29)	
MPCs	PT7 (%)	PTP (%)	PT7 (%)	PTP (%)
1 (Red)	6 (100 %)	0	–	–
2 (Green)	7 (42 %)	10 (58 %)	7 (70 %)	3 (30 %)
3 (Blue)	9 (36 %)	16 (64 %)	9 (47 %)	10 (53 %)
Total	22 (46 %)	26 (54 %)	16 (55 %)	13 (45 %)

## Discussion

We investigated an outbreak of laboratory-confirmed cases of *S. sonnei* in three London boroughs between November 2013 and July 2014 (*n*=52) and found that the majority of cases (92 %) self-identified as, had known links with, or had strong evidence to indicate that they belonged to the OJC. The WGS data enabled us to deduce that the increase in cases observed in the OJC in North London between November 2013 and July 2014 comprised distinct, contemporary outbreak clusters, each containing between 7 and 24 cases, focused around London and Greater Manchester. By reviewing the distribution of demographic characteristics, clinical features and exposures associated with cases within each WGS cluster, we found that MPCs were associated with different regions of England, different areas of London and specific households. This was only apparent following analysis of the WGS data and might reflect heterogeneity in terms of exposures between different groups within the OJC. Interventions could be further tailored to these communities by better understanding such heterogeneity.

Historically, attempting to find common epidemiological factors amongst OJC cases, that were erroneously linked or not linked by phage typing, hindered outbreak investigations by distorting any association between exposure and illness. Findings from this prospective application of WGS during an outbreak of *S. sonnei* among the OJC support those from previous retrospective application of this typing approach for an outbreak amongst the same population in 2011 [5]. Results from the two studies indicated that PTs were not associated with a phylogenetic signal within these clusters and that there were distinct MPCs of more closely related strains and concurrent regional outbreaks occurring in OJCs across the UK. WGS clearly differentiated closely related isolates from background strains of the same phage type circulating in the OJC in the same time frame and illustrated the limitations of phage typing to facilitate outbreak investigations.

Strains from the OJC cases were phylogenetically distinct from the domestically-acquired isolates of *S. sonnei* in the rest of the UK population, and clustered more closely with publicly available sequences from strains of *S. sonnei* isolated in Israel (Fig. S1 and Baker *et al.* [12]). The phylogenetic relationship between the strains indicates that outbreaks in the OJC were not fuelled by ongoing transmission within these communities but by annual or biennial introductions of strains of *S. sonnei* from elsewhere. An outbreak among the OJC in Antwerp was shown to be microbiologically linked to an outbreak in Israel [9]. Global studies indicate that travel back and forth to Israel seeded outbreaks in the OJC in London and Manchester [12]. The temporal pattern of strain introduction and transmission in the OJC in London and Greater Manchester mirrors that described in Tel Aviv and Hafia [23]. Despite this, there is no specific reference to *Shigella* or broader GI infections, upon the UK’s travel health website for travel to Israel [24].

Prior to the implementation of WGS at PHE, public health management of outbreaks of *S. sonnei* were confounded by the lack of a robust typing scheme. With the advent of WGS, we now have access to a molecular typing method that has utility for outbreak detection and investigation. The level of discrimination exhibited by WGS facilitates the identification of clusters of isolates within closely related bacterial populations circulating in the OJC that may be linked to a unique point source or transmission route, thus enabling more appropriate public health response and targeted interventions. Furthermore, these data enable us to track the emergence and dissemination of specific lineages on a global scale. Sharing of such information could result in the timely reinforcement of direct public health messaging to travellers and high-risk domestic populations in the UK, in order to reduce the number of imported infections and mitigate the effects of imported infections and associated outbreaks.

## Data bibliography

1. Dallman TJ, Ashton PA, Jenkins C, Grant K. NCBI Short Read Archive PRJNA248042 (2015).

## Supplementary Data

308 Supplementary File 1
