# Supplementary material for: Whole-genome sequencing revealed concurrent outbreaks of shigellosis in the English Orthodox Jewish Community caused by multiple importations of Shigella sonnei from Israel
Source: Microb Genom. 2018 Mar 27;4(3):e000170. doi: 10.1099/mgen.0.000170 (PMC5885021; doi:10.1099/mgen.0.000170)
Supplement: Supplementary File 1 [file mgen-4-170-s001.pdf]

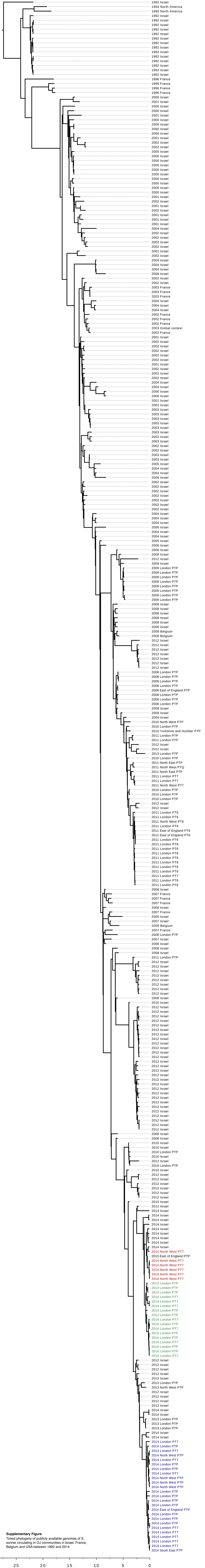

Supplementary Figure
Timed phylogeny of publicly available genomes of S. sonnei circulating in OJ communities in Israel, France, Belgium and USA between 1992 and 2014

Supplementary Table. Sequences used in phylogenetic analyses

Accession number	SNP profile	Geography	Phage Type	Outbreak
ERR572945	1.3.4.218.258.265.281	London	7	YES
SRR5034522	1.3.4.4.17.18.126	London	7	YES
ERR572947	1.3.4.4.17.18.224	London	7	YES
ERR572944	1.3.4.4.17.18.224	London	7	YES
SRR5034522	1.3.4.4.17.18.25	London	7	YES
SRR5034602	1.3.4.4.17.18.283	London	7	YES
ERR572942	1.3.4.4.17.18.51	London	7	YES
SRR5034583	1.3.4.4.17.18.51	London	7	YES
SRR5034525	1.3.4.4.25.26.102	London	7	YES
ERR572943	1.3.4.4.25.26.140	London	7	YES
SRR5034581	1.3.4.4.25.26.34	London	7	YES
SRR5034576	1.3.4.4.25.26.34	London	7	YES
SRR5034534	1.3.4.4.25.26.34	London	7	YES
SRR5034586	1.3.4.4.25.26.47	London	7	YES
ERR572946	1.3.4.4.25.26.73	London	7	YES
SRR5034600	1.3.4.4.25.296.317	London	7	YES
ERR572925	1.3.4.4.17.18.51	London	P	YES
ERR572926	1.3.4.4.17.18.51	London	P	YES
Pending	1.3.4.4.17.18.51	London	P	YES
SRR5034516	1.3.4.4.25.26.118	London	P	YES
SRR5034531	1.3.4.4.25.26.122	London	P	YES
SRR5034533	1.3.4.4.25.26.315	London	P	YES
ERR572920	1.3.4.4.25.26.34	London	P	YES
ERR572921	1.3.4.4.25.26.34	London	P	YES
ERR572922	1.3.4.4.25.26.34	London	P	YES
SRR5034515	1.3.4.4.25.26.34	London	P	YES
SRR5034517	1.3.4.4.25.26.47	London	P	YES
ERR572923	1.3.4.4.25.26.73	London	P	YES
ERR572924	1.3.4.4.25.26.84	London	P	YES
ERR127049	1.3.4.4.6.6.10	North West	6	
ERR127055	1.3.4.4.6.6.11	London	6	
ERR127056	1.3.4.4.6.6.12	London	6	
ERR103471	1.3.4.4.6.6.120	London	6	
ERR127057	1.3.4.4.6.6.13	London	6	
ERR127058	1.3.4.4.6.6.15	London	6	
ERR127061	1.3.4.4.6.6.15	East of England	6	
ERR127059	1.3.4.4.6.6.15	London	6	
ERR127062	1.3.4.4.6.6.15	London	6	
ERR127060	1.3.4.4.6.6.18	East of England	6	
ERR103477	1.3.4.4.6.6.239	London	6	
ERR103475	1.3.4.4.6.6.293	London	6	
ERR103473	1.3.4.4.6.6.304	London	6	
ERR103474	1.3.4.4.6.6.316	London	6	
ERR127053	1.3.4.4.6.6.6	London	6	
ERR103478	1.3.4.4.6.6.79	London	6	
ERR572941	1.3.4.4.17.19.173	North West	7	
Pending	1.3.4.4.17.19.173	North West	7	

SRR5034592	1.3.4.4.17.19.173	North West	7
SRR5034594	1.3.4.4.17.19.173	North West	7
ERR572940	1.3.4.4.17.19.214	North West	7
SRR5034589	1.3.4.4.17.19.289	North West	7
ERR103472	1.3.4.4.6.6.120	London	7
ERR127052	1.3.4.4.7.7.7	London	7
ERR127050	1.3.4.4.7.7.8	London	7
ERR127051	1.3.4.4.7.7.9	North West	7
ERR572876	1.3.4.141.164.169.183	London	P
ERR572913	1.3.4.4.142.146.160	London	P
ERR572911	1.3.4.4.142.146.180	London	P
ERR572935	1.3.4.4.17.18.169	London	P
ERR572937	1.3.4.4.17.18.271	London	P
ERR572918	1.3.4.4.17.18.335	London	P
ERR572938	1.3.4.4.17.18.44	London	P
SRR5034595	1.3.4.4.17.18.51	London	P
SRR5034588	1.3.4.4.17.18.51	London	P
ERR572919	1.3.4.4.17.18.92	London	P
ERR572936	1.3.4.4.17.19.26	East of England	P
ERR572883	1.3.4.4.23.178.192	London	P
ERR572884	1.3.4.4.23.178.192	London	P
ERR572882	1.3.4.4.23.178.215	London	P
ERR572880	1.3.4.4.23.24.128	London	P
ERR572877	1.3.4.4.23.24.148	London	P
ERR572879	1.3.4.4.23.24.267	London	P
ERR572886	1.3.4.4.23.24.290	East of England	P
ERR572878	1.3.4.4.23.24.31	London	P
SRR5034590	1.3.4.4.25.26.157	North West	P
ERR572927	1.3.4.4.25.26.34	East of England	P
SRR5034601	1.3.4.4.25.26.34	South East	P
SRR5034579	1.3.4.4.25.26.73	North West	P
SRR5034519	1.3.4.4.25.26.73	North West	P
SRR5034587	1.3.4.4.25.26.73	North West	P
ERR572909	1.3.4.4.276.283.302	North West	P
ERR572902	1.3.4.4.38.137.149	London	P
ERR572906	1.3.4.4.38.151.165	London	P
ERR572905	1.3.4.4.38.151.170	London	P
ERR572908	1.3.4.4.38.185.199	Yorkshire	P
ERR572903	1.3.4.4.38.39.168	London	P
ERR572901	1.3.4.4.38.39.48	London	P
ERR572916	1.3.4.4.4.4.4	London	P
ERR572933	1.3.4.4.40.117.129	North West	P
ERR572928	1.3.4.4.40.126.138	London	P
ERR572907	1.3.4.4.40.191.206	London	P
ERR572904	1.3.4.4.40.191.313	London	P
ERR572930	1.3.4.4.40.41.134	London	P
ERR572932	1.3.4.4.40.41.249	London	P
ERR572934	1.3.4.4.40.41.50	London	P
ERR572914	1.3.4.4.7.82.161	North East	P
ERR1364236	1.3.4.4.7.82.255	North East	P

ERR572892	1.3.4.4.78.79.100	London	P
ERR572895	1.3.4.4.78.79.100	London	P
ERR572898	1.3.4.4.78.79.100	London	P
ERR572899	1.3.4.4.78.79.177	London	P
ERR572894	1.3.4.4.78.79.179	London	P
ERR572897	1.3.4.4.78.79.228	London	P
ERR572896	1.3.4.4.78.79.229	London	P
ERR572893	1.3.4.4.78.79.90	London	P
ERR572929	1.3.4.4.84.86.97	London	P
Pending	1.3.4.4.7.82.93	North West	Q
ERR190896	1.168.250.538.795.888.1211	Israel	
ERR563030	1.172.264.564.839.935.1258	Israel	
ERR563022	1.172.264.564.839.935.1314	Israel	
ERR190900	1.174.266.567.852.950.1275	Israel	
ERR190898	1.3.222.506.729.819.1139	Israel	
ERR563027	1.3.224.508.735.825.1146	Israel	
ERR558529	1.3.224.508.735.825.1149	Israel	
ERR558530	1.3.224.508.735.825.1149	Israel	
ERR558531	1.3.224.508.735.825.1149	Israel	
ERR563025	1.3.224.508.735.825.1149	Israel	
ERR563028	1.3.224.508.735.825.1149	Israel	
ERR558522	1.3.224.508.735.825.1280	Israel	
ERR558525	1.3.224.508.735.825.1280	Israel	
ERR563024	1.3.224.508.735.825.1280	Israel	
ERR558527	1.3.224.508.735.825.1281	Israel	
ERR558528	1.3.224.508.735.825.1297	Israel	
ERR563026	1.3.224.508.735.825.1320	Israel	
ERR558526	1.3.224.508.735.825.1461	Israel	
ERR558524	1.3.224.508.735.825.1468	Israel	
ERR042792	1.3.224.518.758.849.1170	North America	
ERR042793	1.3.224.605.935.1040.1370	North America	
ERR042791	1.3.230.516.752.1041.1371	France	
ERR042790	1.3.230.516.752.843.1164	France	
ERR042788	1.3.256.549.817.911.1234	France	
ERR558523	1.3.272.574.863.961.1286	Israel	
ERR042795	1.3.293.604.934.1039.1369	North America	
ERR190855	1.3.3.3.912.1017.1346	Israel	
ERR042789	1.3.326.642.1034.1143.1479	France	
ERR319246	1.3.4.4.1012.1120.1456	Israel	
ERR047220	1.3.4.4.1018.1127.1463	Israel	
ERR190893	1.3.4.4.1020.1129.1465	Israel	
ERR190895	1.3.4.4.1022.1131.1467	Israel	
ERR042782	1.3.4.4.1027.1136.1472	France	
ERR563023	1.3.4.4.17.19.1144	Israel	
ERR563018	1.3.4.4.17.19.1205	Israel	
ERR563032	1.3.4.4.17.19.1259	Israel	
ERR563016	1.3.4.4.17.19.1365	Israel	
ERR563031	1.3.4.4.17.19.1445	Israel	
ERR563020	1.3.4.4.17.990.1317	Israel	
ERR190894	1.3.4.4.23.1000.1328	Israel	

ERR190897	1.3.4.4.23.24.1309	Israel
ERR319255	1.3.4.4.4.1006.1334	Israel
ERR211146	1.3.4.4.4.1006.1417	Israel
ERR319268	1.3.4.4.4.4.1174	Israel
ERR319245	1.3.4.4.4.4.1269	Israel
ERR211158	1.3.4.4.4.4.1299	Israel
ERR190905	1.3.4.4.4.4.1421	Israel
ERR211150	1.3.4.4.4.4.1455	Israel
ERR190913	1.3.4.4.4.4.1480	Israel
ERR319214	1.3.4.4.40.1010.1338	Israel
ERR039524	1.3.4.4.40.191.1324	Israel
ERR039525	1.3.4.4.40.191.1386	Israel
ERR039529	1.3.4.4.40.191.1390	Israel
ERR039527	1.3.4.4.40.886.1209	Israel
ERR319205	1.3.4.4.40.886.1255	Israel
ERR319252	1.3.4.4.40.886.1262	Israel
ERR319206	1.3.4.4.40.886.1274	Israel
ERR319253	1.3.4.4.40.886.1274	Israel
ERR190910	1.3.4.4.40.886.1337	Israel
ERR190914	1.3.4.4.40.886.1337	Israel
ERR319204	1.3.4.4.40.886.1442	Israel
ERR211151	1.3.4.4.40.886.1447	Israel
ERR211154	1.3.4.4.40.886.1447	Israel
ERR047221	1.3.4.4.40.892.1215	Israel
ERR190812	1.3.4.4.720.810.1130	Israel
ERR190784	1.3.4.4.721.1078.1411	Israel
ERR190785	1.3.4.4.721.1080.1413	Israel
ERR190787	1.3.4.4.721.1081.1414	Israel
ERR190800	1.3.4.4.721.1106.1440	Israel
ERR190795	1.3.4.4.721.1118.1453	Israel
ERR190813	1.3.4.4.721.811.1131	Israel
ERR190816	1.3.4.4.721.811.1143	Israel
ERR190797	1.3.4.4.721.811.1449	Israel
ERR190819	1.3.4.4.721.813.1133	Israel
ERR190817	1.3.4.4.721.813.1138	Israel
ERR190818	1.3.4.4.721.813.1138	Israel
ERR190820	1.3.4.4.721.813.1138	Israel
ERR045194	1.3.4.4.721.813.1159	Israel
ERR190843	1.3.4.4.721.813.1185	Israel
ERR190798	1.3.4.4.721.813.1265	Israel
ERR190794	1.3.4.4.721.813.1268	Israel
ERR190792	1.3.4.4.721.813.1271	Israel
ERR190799	1.3.4.4.721.813.1290	Israel
ERR190780	1.3.4.4.721.813.1293	Israel
ERR190783	1.3.4.4.721.813.1293	Israel
ERR190791	1.3.4.4.721.813.1293	Israel
ERR190823	1.3.4.4.721.813.1294	Israel
ERR190779	1.3.4.4.721.813.1321	Israel
ERR190801	1.3.4.4.721.813.1398	Israel
ERR190793	1.3.4.4.721.813.1454	Israel

ERR190775	1.3.4.4.721.813.1462	Israel
ERR042777	1.3.4.4.721.898.1221	France
ERR190786	1.3.4.4.721.905.1228	Israel
ERR190788	1.3.4.4.721.906.1229	Israel
ERR190789	1.3.4.4.721.920.1243	Israel
ERR190796	1.3.4.4.721.942.1266	Israel
ERR042786	1.3.4.4.725.1135.1471	France
ERR190847	1.3.4.4.725.815.1135	Israel
ERR190850	1.3.4.4.725.815.1348	Israel
ERR045199	1.3.4.4.725.831.1152	Israel
ERR190860	1.3.4.4.725.831.1291	Israel
ERR042783	1.3.4.4.725.831.1326	France
ERR047212	1.3.4.4.725.831.1343	Israel
ERR190859	1.3.4.4.725.831.1353	Israel
ERR042784	1.3.4.4.725.831.1469	France
ERR190861	1.3.4.4.725.831.1474	Israel
ERR190846	1.3.4.4.725.840.1161	Israel
ERR190840	1.3.4.4.725.840.1202	Israel
ERR190844	1.3.4.4.725.840.1384	Israel
ERR190857	1.3.4.4.725.856.1178	Israel
ERR319216	1.3.4.4.727.817.1137	Israel
ERR190854	1.3.4.4.730.820.1140	Israel
ERR190838	1.3.4.4.730.915.1238	Israel
ERR190849	1.3.4.4.730.915.1256	Israel
ERR190842	1.3.4.4.730.915.1388	Israel
ERR319190	1.3.4.4.731.821.1141	Israel
ERR319186	1.3.4.4.731.821.1157	Israel
ERR319258	1.3.4.4.731.821.1157	Israel
ERR319256	1.3.4.4.731.821.1158	Israel
ERR319254	1.3.4.4.731.821.1160	Israel
ERR319224	1.3.4.4.731.821.1188	Israel
ERR319222	1.3.4.4.731.821.1189	Israel
ERR319185	1.3.4.4.731.821.1220	Israel
ERR319189	1.3.4.4.731.821.1222	Israel
ERR190908	1.3.4.4.731.821.1272	Israel
ERR190917	1.3.4.4.731.821.1272	Israel
ERR190906	1.3.4.4.731.821.1277	Israel
ERR190909	1.3.4.4.731.821.1285	Israel
ERR211153	1.3.4.4.731.821.1308	Israel
ERR211157	1.3.4.4.731.821.1312	Israel
ERR211155	1.3.4.4.731.821.1313	Israel
ERR190916	1.3.4.4.731.821.1339	Israel
ERR319198	1.3.4.4.731.821.1341	Israel
ERR319191	1.3.4.4.731.821.1352	Israel
ERR319223	1.3.4.4.731.821.1361	Israel
ERR319188	1.3.4.4.731.821.1375	Israel
ERR319187	1.3.4.4.731.821.1400	Israel
ERR190907	1.3.4.4.731.821.1427	Israel
ERR211156	1.3.4.4.731.821.1457	Israel
ERR190915	1.3.4.4.731.821.1475	Israel

ERR319215	1.3.4.4.731.821.1476	Israel
ERR190911	1.3.4.4.731.821.1478	Israel
ERR319248	1.3.4.4.731.962.1287	Israel
ERR190858	1.3.4.4.732.822.1142	Israel
ERR190862	1.3.4.4.732.822.1292	Israel
ERR190851	1.3.4.4.732.865.1187	Israel
ERR190852	1.3.4.4.736.826.1147	Israel
ERR190778	1.3.4.4.744.1123.1459	Israel
ERR190777	1.3.4.4.744.1123.1464	Israel
ERR190774	1.3.4.4.744.835.1156	Israel
ERR190745	1.3.4.4.751.842.1163	Israel
ERR190746	1.3.4.4.751.842.1171	Israel
ERR190748	1.3.4.4.751.842.1172	Israel
ERR190751	1.3.4.4.751.842.1217	Israel
ERR190757	1.3.4.4.751.842.1218	Israel
ERR190753	1.3.4.4.751.842.1224	Israel
ERR190754	1.3.4.4.751.842.1231	Israel
ERR190749	1.3.4.4.751.842.1263	Israel
ERR190760	1.3.4.4.751.842.1267	Israel
ERR190750	1.3.4.4.751.842.1373	Israel
ERR190756	1.3.4.4.751.842.1374	Israel
ERR190752	1.3.4.4.751.842.1376	Israel
ERR190759	1.3.4.4.751.842.1377	Israel
ERR190755	1.3.4.4.751.842.1389	Israel
ERR190771	1.3.4.4.751.842.1422	Israel
ERR190758	1.3.4.4.751.902.1225	Israel
ERR190770	1.3.4.4.751.996.1323	Israel
ERR190802	1.3.4.4.751.996.1323	Israel
ERR319197	1.3.4.4.753.844.1165	Israel
ERR042779	1.3.4.4.755.1069.1402	France
ERR042780	1.3.4.4.755.1100.1434	France
ERR042778	1.3.4.4.755.846.1167	France
ERR042776	1.3.4.4.756.1034.1363	France
ERR042794	1.3.4.4.756.847.1168	France
ERR025767	1.3.4.4.756.847.1315	Israel
ERR042775	1.3.4.4.756.896.1219	France
ERR047217	1.3.4.4.757.848.1169	Israel
ERR190803	1.3.4.4.768.859.1181	Israel
ERR190845	1.3.4.4.771.862.1184	Israel
ERR190809	1.3.4.4.773.864.1186	Israel
ERR190806	1.3.4.4.777.868.1190	Israel
ERR190805	1.3.4.4.777.868.1391	Israel
ERR047216	1.3.4.4.78.79.1345	Israel
ERR047218	1.3.4.4.78.832.1153	Israel
ERR190804	1.3.4.4.781.1049.1379	Israel
ERR190810	1.3.4.4.781.1055.1385	Israel
ERR190808	1.3.4.4.781.873.1195	Israel
ERR563017	1.3.4.4.784.876.1198	Israel
ERR563015	1.3.4.4.786.878.1200	Israel

ERR449080	1.3.4.4.818.912.1235	Belgium
ERR449083	1.3.4.4.818.912.1396	Belgium
ERR449084	1.3.4.4.819.913.1236	Belgium
ERR190839	1.3.4.4.822.1085.1418	Israel
ERR190836	1.3.4.4.822.916.1239	Israel
ERR190837	1.3.4.4.822.916.1381	Israel
ERR211148	1.3.4.4.829.923.1246	Israel
ERR319208	1.3.4.4.829.923.1276	Israel
ERR319259	1.3.4.4.829.923.1329	Israel
ERR319257	1.3.4.4.829.923.1333	Israel
ERR190767	1.3.4.4.841.937.1261	Israel
ERR190761	1.3.4.4.843.1087.1420	Israel
ERR190768	1.3.4.4.843.940.1264	Israel
ERR190902	1.3.4.4.851.948.1273	Israel
ERR047214	1.3.4.4.856.1016.1344	Israel
ERR190904	1.3.4.4.856.954.1279	Israel
ERR190825	1.3.4.4.866.964.1289	Israel
ERR190824	1.3.4.4.866.964.1437	Israel
ERR190835	1.3.4.4.866.964.1438	Israel
ERR190899	1.3.4.4.885.983.1310	Israel
ERR190776	1.3.4.4.898.997.1325	Israel
ERR042785	1.3.4.4.902.1002.1330	France
ERR190912	1.3.4.4.906.1008.1336	Israel
ERR319220	1.3.4.4.906.1008.1372	Israel
ERR190747	1.3.4.4.915.1020.1349	Israel
ERR045200	1.3.4.4.917.1022.1351	Israel
ERR047213	1.3.4.4.922.1027.1356	Israel
ERR047219	1.3.4.4.926.1031.1360	Israel
ERR563014	1.3.4.4.931.1036.1366	Israel
ERR563033	1.3.4.4.931.1036.1446	Israel
ERR563019	1.3.4.4.933.1038.1368	Israel
ERR190807	1.3.4.4.961.1066.1399	Israel
ERR190848	1.3.4.4.974.1079.1412	Israel
ERR190762	1.3.4.4.978.1083.1416	Israel
ERR190769	1.3.4.4.980.1086.1419	Israel
ERR190903	1.3.4.4.983.1089.1423	Israel
ERR319207	1.3.4.4.984.1090.1424	Israel
ERR190901	1.3.4.4.986.1092.1426	Israel
ERR211149	1.3.4.4.989.1095.1429	Israel
ERR319249	1.3.4.4.993.1099.1433	Israel
ERR190790	1.3.4.4.999.1105.1439	Israel
ERR319250	1.3.4.513.742.833.1154	Israel
ERR319221	1.3.4.513.742.833.1173	Israel
ERR319203	1.3.4.513.742.833.1253	Israel
ERR319247	1.3.4.513.742.833.1270	Israel
ERR319251	1.3.4.513.742.833.1270	Israel
ERR319213	1.3.4.513.742.981.1307	Israel
ERR039528	1.3.4.546.813.907.1230	Israel
ERR211147	1.3.4.563.838.934.1257	Israel
